# Reducing Cardiac Injury during ST-Elevation Myocardial Infarction: A Reasoned Approach to a Multitarget Therapeutic Strategy

**DOI:** 10.3390/jcm10132968

**Published:** 2021-07-01

**Authors:** Alessandro Bellis, Giuseppe Di Gioia, Ciro Mauro, Costantino Mancusi, Emanuele Barbato, Raffaele Izzo, Bruno Trimarco, Carmine Morisco

**Affiliations:** 1 Unità Operativa Complessa Cardiologia con UTIC ed Emodinamica—Dipartimento Emergenza Accettazione, Azienda Ospedaliera “Antonio Cardarelli”, Via A. Cardarelli n. 9, 80131 Napoli, Italy; abellis82@vodafone.it (A.B.); ciro.mauro1957@gmail.com (C.M.); 2 Cardiac Catheterization Laboratory, Montevergine Clinic, Via M. Malzoni, 83013 Mercogliano, Italy; di-gioia@libero.it; 3 Dipartimento di Scienze Biomediche Avanzate, Università FEDERICO II, Via S. Pansini n. 5, 80131 Napoli, Italy; costantino.mancusi@unina.it (C.M.); emanuele.barbato@unina.it (E.B.); rafizzo@unina.it (R.I.); trimarco@unina.it (B.T.); 4 Cardiovascular Center Aalst, OLV Clinic, 9300 Aalst, Belgium

**Keywords:** left ventricular remodeling, extracellular matrix, remote ischemic conditioning, coronary microvascular obstruction, primary percutaneous coronary intervention

## Abstract

The significant reduction in ‘ischemic time’ through capillary diffusion of primary percutaneous intervention (pPCI) has rendered myocardial-ischemia reperfusion injury (MIRI) prevention a major issue in order to improve the prognosis of ST elevation myocardial infarction (STEMI) patients. In fact, while the ischemic damage increases with the severity and the duration of blood flow reduction, reperfusion injury reaches its maximum with a moderate amount of ischemic injury. MIRI leads to the development of post-STEMI left ventricular remodeling (post-STEMI LVR), thereby increasing the risk of arrhythmias and heart failure. Single pharmacological and mechanical interventions have shown some benefits, but have not satisfactorily reduced mortality. Therefore, a multitarget therapeutic strategy is needed, but no univocal indications have come from the clinical trials performed so far. On the basis of the results of the consistent clinical studies analyzed in this review, we try to design a randomized clinical trial aimed at evaluating the effects of a reasoned multitarget therapeutic strategy on the prevention of post-STEMI LVR. In fact, we believe that the correct timing of pharmacological and mechanical intervention application, according to their specific ability to interfere with survival pathways, may significantly reduce the incidence of post-STEMI LVR and thus improve patient prognosis.

## 1. Introduction

In recent decades, most of the efforts in the treatment of ST-elevation myocardial infarction (STEMI) have been focused on the organization of public health systems aimed at guaranteeing the prompt coronary revascularization of the culprit artery [1,2,3] and developing pharmacological treatments for further preservation of the coronary blood flow [4,5,6,7].

Although the reduction in ‘ischemia-time’ through an early primary percutaneous intervention (pPCI; within 2 h since symptoms onset) has significantly improved the outcomes of STEMI patients, reperfusion inflicts metabolic injuries that are both reversible, such as stunning [8], and irreversible and manifest, such as increased infarct size (IS) that is strictly dependent on the coronary microvascular obstruction (CMVO). In fact, while the ischemic damage increases with the severity and the duration of blood flow reduction, reperfusion injury reaches its maximum with a moderate amount of ischemic injury [9]. This phenomenon is called myocardial ischemia-reperfusion injury (MIRI) [10,11] and leads to the development of post-infarction left ventricular remodeling (LVR) [12,13,14]. The arrhythmias and heart failure (HF) deriving from LVR negatively affect the short- and long-term prognosis of patients with STEMI and, at the same time, highlight the need to integrate current strategies with additional therapies [15].

Many cardioprotective strategies against MIRI have been proposed [16]. However, so far, none of these have shown an improvement in the clinical outcomes of STEMI patients. An important reason for the weak and inconsistent results obtained in these patients may be the presence of multiple partially redundant mechanisms of cell death during ischemia–reperfusion, whose relative importance may change depending on the conditions. Therefore, it is recognized that it is important to consider a multitarget cardioprotective therapy, defined as additive or synergistic cardioprotective agents or interventions directed towards distinct targets with different application timings (before, during, or after pPCI) [17].

In this review, (a) we discuss the pathogenic mechanisms that are responsible for the development of LVR and microcirculation injury; (b) we describe the conventional and emerging pharmacological treatments, as well as the mechanical interventions, that have been shown to enhance cardioprotection; and (c) we try to design a randomized clinical trial aimed at evaluating the effects of a reasoned multitarget therapeutic strategy on the prevention of post-STEMI LVR.

## 2. Mechanisms of Post-Infarction LVR

LVR is a maladaptive process, which leads to left ventricle (LV) hypertrophy and HF. Several hemodynamic, anthropomorphic, and metabolic abnormalities (such as arterial hypertension, obesity, diabetes mellitus, cardiac valves disease, chronic kidney disease, and ischemic heart disease) are responsible for the development of LVR [18,19,20,21,22,23]. Specifically, post-infarction LVR is due to changes to the geometric profile of LV, and is defined as an increase ≥20% or ≥12% of the indexed LV end-diastolic volume (iLVEDV) detected with echocardiography or with magnetic resonance, respectively, six months after an acute myocardial infarction (AMI) [14,24].

**Figure 1:**
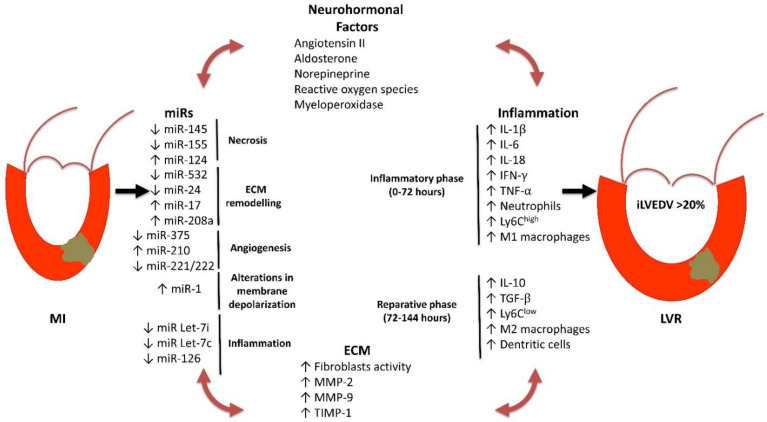
Schematic representation of the different mechanisms involved in the development of post-STEMI LVR. ECM: extracellular matrix; iLVEDV: indexed left ventricular end-diastolic volume; LVR: left ventricular remodeling; STEMI: ST elevation myocardial infarction; miRs: micro-RNAs; IL: interleukin; IFN-γ: interferon-γ; TNF-α: tumor necrosis factor-α; Ly6c: lymphocyte 6 cytotoxic; TGF-β: tumor growth factor-β; MMPs: metalloproteinases; TIMPs: tissue inhibitor of metalloproteinases. ↑ indicates an increase; ↓ indicates a decrease.

Development of post-infarction LVR is a complex and multifactorial process that involves several determinants including size and localization of necrosis, timing and efficacy of reperfusion, local and systemic inflammation, changes of homeostasis of extra-cellular matrix (ECM), redox imbalance, reparative processes, sustained neuro-hormonal activation (norepinephrine, angiotensin II, aldosterone), CMVO, and dysregulation of transcriptional activities [25,26,27,28,29]. It must be underlined that all these determinants act in concert in the pathogenesis of LVR, resulting in a vicious circle that ultimately compromises the morphological and functional characteristics of the infarcted heart (Figure 1).

In recent years, the attention of research has been focused on the role of non-coding RNAs (ncRNAs), inflammation, ECM remodeling, and CMVO in the pathogenesis of LVR.

### 2.1. ncRNAs

The family of ncRNAs includes various types of RNAs, which, in contrast to messenger RNAs (mRNAs), are not translated into proteins. Most important types of ncRNAs involved in development of post-AMI LVR are micro-RNAs (miRs), long non-coding RNAs (lncRNAs), and circular RNAs (circRNAs).

The miRs are small, single-stranded fragments of non-coding RNA with a length of 22–24 nucleotides regulating the gene expression. The miRs play a key role in the control of several physiologic processes, such as the differentiation of immune, hematopoietic, and skeletal muscle cells, and the regulation of angiogenesis, neurogenesis, and immune response. In general, miRs act as silencing genes at the transcriptional and post-transcriptional level [30]. The role of miRs has also been documented in cardiovascular (CV) diseases, since they are involved in the regulation of myocardial apoptosis and fibrosis, endothelial growth, and cell differentiation [31,32,33]. Nowadays, miRs are used as biomarkers for the stratification of CV prognosis [34]. In humans, several miRs (miR-145, miR-155, miR-124) have been found to be markers of myocardial ischemia and have been demonstrated to correlate with the extension of AMI [35,36,37].

In addition, experimental data demonstrate that miRs play a mechanistic role in the development of LVR (Table 1). In particular, they are involved in the regulation of the cardiac fibrosis, the differentiation of mesenchymal stem cells, the regulation of cell death/survival, and electric conduction [26,38]. The expression of miR-24 was reduced in an experimental model of AMI. Notably, miR-24 decreases the expression of transforming growth factor (TGF)-β1 that is implied in cardiac fibrosis following AMI. Down-regulation of miR-24 was associated with the development of extensive fibrosis in the area at risk, reduced cardiac function, and enhanced expression of fibronectin, type 1 collagen, and TGF-β1. The enhanced expression of miR-24, obtained with myocardial injection of a lentiviral vector, completely restored the maladaptive phenotype induced by AMI [39]. Similarly, miR-532 interferes with the development of LVR by reducing the post-infarction myocardial fibrosis. This phenomenon is mediated by the inhibition of prss23 serine protease expression, which stimulates the synthesis of ECM proteins from the cardiac fibroblasts. In addition, miR-532 inhibits the transformation of endothelial cells in cardiac fibroblasts [40]. The miR-208a is essential for the expression of the genes involved in cardiac hypertrophy and fibrosis, such as endoglin [41]. In particular, miR-208a activates endoglin expression and may result in cardiac fibrosis in rats with AMI [42]. A further miR involved in the post-infarction ECM remodeling, but with an opposing role, is called miR-17. The expression of miR-17 was upregulated in experimental AMI. It was found to be associated with a decrease in tissue inhibitors of metalloproteinases (TIMPs) 1 and 2 and enhanced proteolytic activity of metalloproteinase (MMP)-9. These abnormalities increased ECM degradation and were responsible for a severe impairment of LV function. Inhibition of endogenous miR-17 by antagomir prevented the LVR after AMI [43].

The angiogenesis and reparative processes represent further pathogenic mechanisms with a critical role in LVR and are also finely regulated by miRs. Experimental AMI enhances miR-375, which negatively interferes with the reparative processes through the inhibition of the PDK1/PI3K/Akt pathway. The knockdown of miR-375 ameliorates the survival of marrow-derived angiogenic progenitor cells, favoring the neo-angiogenesis of injured myocardium. This fact contributes to improving the reparative processes and to preserving cardiac function [44]. Similarly, the intracoronary injection of antagomir-92a, in experimental AMI, inhibits miR-92a in the area at risk, favoring neo-angiogenesis and preventing LVR [45]. It has been demonstrated that miR-210 regulates angiogenesis and is upregulated by ischemia-reperfusion stress. However, an intravenous injection of lentivirus encoding for miR-210 agonists induces an upregulation of miR-210, which increases the microvessel density in the area at risk and, at the same time, preserves LV contractility in experimental AMI [46].

The miRs can also interfere with LVR by modulating cardioprotective signaling. In particular, it has been reported that cardiac overexpression of miR-21 in transgenic mice confers resistance to ischemia-reperfusion stress through the activation of the anti-apoptotic pathway [47]. On the contrary, the inhibition of miR-21 activity increases the reactive oxygen species (ROS)-mediated cell death of cardiomyocytes [48]. The overexpression of miR-21, obtained by adenovirus infection of rat hearts, was able to decrease IS by 29% and reduce the occurrence of LVR [49].

Finally, miRs may be implied in the regulation of membrane depolarization and cardiac electric conduction during cardiac ischemia. Several reports have shown that miR-1 is up-regulated in heart tissue after AMI [50]. The gap junction protein connexin43 (Cx43) is a recognized target of miR-1 and is expressed in cardiomyocytes and cardiac fibroblasts [51]. Cx43 participates in intercellular communication between adjacent cardiomyocytes [52]. Higher miR-1 levels may decrease Cx43 expression, thereby affecting cardiac depolarization and favoring ischemic arrhythmias.

Less well known is the role of lncRNAs in the development of post-AMI LVR. In fact, although lncRNAs are reported to largely participate in myocardial autophagy (Neat1, AK139328, APF, CAIF, and AK088388), apoptosis (CARL, MALAT1, HOTAIR, UCA1, and XIST), and necrosis (NRF and H19), no clinical trial has been published [53]. Similarly, there are few relevant data about the role of circRNAs in the development of post-AMI LVR. Some studies have shown that a type of myocardial infarction-associated circular RNA (MICRA) is mainly expressed in peripheral blood cells [54,55]. Compared with healthy controls, MICRA expression in the peripheral blood cells of AMI patients was significantly decreased [55]. Multivariate analysis showed that MICRA was a strong predictor of significantly decreased LV function [55].

### 2.2. Inflammation

Definitively, STEMI is the result of an inflammatory process accounting for the development and leading to the vulnerability of atherosclerotic plaque [56,57]. Inflammation time-dependently recruits different cells, mediators, and receptors. The inflammatory response in STEMI represents a defense mechanism aimed at preventing the infection of the injured tissue; moreover, it also plays a key role in repairing the necrotic tissue. The hypoxia-reperfusion stress, through the local generation of ROS, triggers a complex and multifaceted response including the expression and activation of adhesion molecules, chemokines, and cytokines. Together, these mediators recruit the inflammatory cells in the injured area.

Neutrophils are the first cell population activated in the infarcted area. These cells, through their degranulation, digest several products released from the necrotic cells, and of ECM. In addition, they contribute to further amplifying local inflammation through the release of ROS, myeloperoxidase, cytokines, such as interleukin (IL)-1β, IL-18, and IL-6, interferon (INF)-γ, and tumor necrosis factor (TNF)-α, which attract and activate monocytes and lymphocytes in the infarcted area. The cytokines finely regulate the interplay among the different immune competent cells. This inflammatory activity characterizes the first 48–72 h of the STEMI acute phase.

Subsequently, there is a progressive attenuation of local inflammation and the reparative phase begins. The main feature of this phase is the change of phenotype of cell population in the infarcted area. In particular, there is a gradual replacement of Ly6C^high^ and M1 macrophages with Ly6C^low^ and M2 macrophages. Such an event is associated with a reduction in pro-inflammatory, and increase in anti-inflammatory and pro-fibrotic cytokines, such as lL-10 and TGF-β. In this phase, the injured area is also infiltrated by dendritic cells, which contribute to resolving the local inflammation and promote scar formation and angiogenesis [58]. The ultimate effect of this phase consists of the inhibition of proteases and the increased expression and activity of TIMPs, resulting in the termination of degradation and the start of the healing processes [59].

There is clinical and experimental evidence showing that systemic and local inflammation are associated with LVR; however, it is still unclear whether this association also has a pathogenic relevance. Basically, all conventional CV risk factors are characterized by a low grade of vascular inflammation [60,61,62,63], which accounts for the pathogenesis of major CV events. Therefore, patients with AMI have a chronic systemic inflammatory state. On the other hand, there is clear evidence that LV dysfunction and HF are associated with an increase in biomarkers of inflammation such as C-reactive protein, adhesion molecules, and cytokines [64,65,66]. Although the results of randomized placebo-controlled clinical studies are not encouraging because they lack the benefit of targeted anti-inflammatory therapy in the prevention on LVR and HF [67,68,69,70], there are experimental findings showing the mechanistic role of inflammation in the pathogenesis of post-infarction LVR. In particular, in genetically engineered mice with an ablation of the gene encoding for the receptor of IL-1 (IL-1RI^−/−^), a lower infiltration of neutrophils and macrophages and a reduced expression of chemokines and cytokines were detected in the area at risk, after experimental AMI, compared to wild type mice. This was associated with reduced fibrotic response and LVR [71]. Similar results were achieved with the pharmacological block of the IL-1 receptor, obtained with the recombinant human IL-1 receptor antagonist (Anakinra). In particular, immediate and delayed administration of Anakinra in an experimental model of AMI determined a significant reduction in apoptosis in the area at risk, compared to the sham operated control animals. This was associated with a more favorable LVR [72]. Finally, the plasma levels of IL-1β detected two months after STEMI in patients treated with pPCI were found to be predictive of adverse LVR [73]. Altogether, these data indicate the mechanistic role of local inflammation in the pathogenesis of LVR, and suggest the further study of the antagonism of cytokine pathway as a potential therapeutic target in the prevention of post-infarction LVR.

### 2.3. ECM Homeostasis

The myocardial fibrosis following AMI is a feature of LVR, and can be defined as the expansion of the LV interstitium due to net accumulation of ECM [28]. It is the result of the cross-talk among ECM, transcriptional and post-transcriptional factor activity, local and systemic inflammation, endocrine/paracrine stimulation, and LV wall stress. Cardiac ECM is the structural scaffold for cardiac myocytes and contains different structural proteins such as type I and III collagen, elastin, laminin, and a cellular component (fibroblasts and inflammatory cells, including mast cells and macrophages). Moreover, ECM is a dynamic tissue whose homeostasis is finely regulated by zinc-dependent proteolytic activity of MMPs and TIMPs, and by the integrins. These latter are cell membrane proteins and act as mechanoreceptors, allowing the intracellular transduction of mechanical stress [74,75]. The replacement fibrosis following AMI is the result of an imbalance between the synthesis and degradation of ECM. This phenomenon, at the beginning, is reparative, thereby allowing scar formation in the infarcted area. However, the progression of this process leads to changes in ECM composition not only in the infarcted zone, but also in the LV remote areas. Together these abnormalities account for the impairment of LV compliance, structure, and geometry, which represent the basis of LVR.

The fibroblasts play a key role in the ECM remodeling; indeed, following the ischemic stress, they rapidly differentiate into myofibroblasts [76]. These cells synthetize collagen and other ECM proteins. The inadequate control of this process results in an excess of ECM deposition, which represents the first step of LV chamber enlargement.

The enzymatic component of ECM also plays a key role in the pathogenesis of LVR. The expression and activity of MMPs and TIMPs during AMI are finely regulated by transcriptional factors, proteolytic activation, and endogenous inhibition. In particular, experimental evidence showed that expression levels of MMP-2, MMP-8, and MMP-13 increase in the infarcted area, whereas MMP-1 and MMP-7 levels significantly decrease. This evidence has been confirmed in humans. Furthermore, in subjects with AMI, it has also been demonstrated that dysregulation of MMPs and TIMPs has a role in the pathogenesis of LVR. In fact, during the early phases, an upregulation of TIMP-1 and MMP-9 has been recorded [77,78]. The persistence of high MMP-9 expression was found to be associated with a higher risk of LVR. In addition, the ratio between MMP-9/TIMP-4 was found to better correlate with LVR [79]. Thus, rather than the variation of the single MMPs or TIMPs, the ratio of MMPs/TIMPs is prominent to better predict the development of LVR.

The concept that the activity of MMPs plays a mechanistic role in post infarction LVR is supported by the evidence that transgenic mice with a knockout gene encoding for MMP-9 (MMP-9 KO) showed significantly smaller increases in both end-diastolic and end-systolic LV diameters 15 days after experimental AMI. This finding was associated with less collagen accumulation in the LV and lower infiltration in the infarcted area of macrophages compared to the control mice. Moreover, in MMP-9 KO mice, an enhanced expression of MMP-2, MMP-13, and TIMP-1 was found [80]. Similar results were obtained in transgenic MMP-2 KO mice [81].

Notably, both the cellular and enzymatic component of ECM are regulated by inflammation. In particular, it has been documented that, in the inflammatory phase of MI, the M1 macrophages release the enzymes with proteolytic activity, such as MMPs and cathepsin, which contribute to ECM remodeling. In addition, it has been demonstrated that the migration of fibroblasts and their transformation into myofibroblats is regulated by pro-inflammatory cytokines such as TNF-α, IL-6, and IL-1β. The recruitment of fibroblasts also persists in the reparative phase under the control of TGF-β. Thus, the interplay between ECM and inflammation finely regulates the fibrotic process following AMI [28].

### 2.4. Coronary Microvascular Dysfunction

Although in recent decades much progresses has been made in our understanding of the genetic basis of platelet function and the mechanisms of antiplatelet therapy resistance [82,83,84], as well as the genetic background of AMI and its complications [85,86,87], coronary microvascular dysfunction still affects almost 50% of patients with AMI, even after prompt epicardial recanalization of the infarct-related artery by pPCI and optimal antiplatelet therapy [88].

The main determinant of coronary microvascular dysfunction is the CMVO that is associated with a four-fold increase in the risk of death and an eight-fold increase in the risk of future hospitalization for HF [89]. Risk factors for the development of CMVO are hypertension, ageing, insulin resistance and diabetes, hyperlipidemia, and chronic inflammatory diseases [90].

Multiple pathophysiological mechanisms lead to the development of CMVO, including (1) ischemia- and reperfusion-related injury, (2) distal embolization, and (3) intra-myocardial hemorrhage (IMH).

(1) Ischemia-reperfusion damages the myocardium through edema (both intracellular and interstitial), impaired vasomotility, and intravascular cell aggregates. Intracellular and interstitial edema quickly develops after coronary occlusion [91,92]. Cardiomyocytes and endothelial cells intracellular edema is the consequence of the energetic deficit and the impairment of energy-dependent ion pumps, whereas interstitial edema develops due to the increased interstitial osmolarity from the release of ions and catabolites, and the dysfunction of the endothelial barrier [93]. This latter is composed of endothelial cells, glycocalyx, and pericytes. Endothelial dysfunction contributes to edema, since adenosine (Ade) release enhances cytoskeletal derangement, followed by hyper-contracture and gap formation [94]. Another determinant of edema is represented by the degradation of the glycocalyx mediated by TNF-α promoting platelet and leukocytes adherence and aggregation [95,96,97]. During reperfusion, the rapid washout of osmotically active substances from the intravascular space increases edema formation. Notably, interstitial myocardial edema compresses capillaries and small vessels, further decreasing flow in these already dysfunctional territories.
  Dysfunctional vasomotility mostly depends on the impairment of endothelium-mediated vasodilatation caused by the disruption of the endothelial barrier [98]. The amount of impairment is proportional to the ischemic insult, being more pronounced in AMI than in chronic hypo-perfusion. Of note, during ischemia-reperfusion, the myocardium remains susceptible to vasoconstrictor stimuli, such as the release of α-adrenergic molecules, serotonin, and thromboxane [99]. Furthermore, the stasis and the increased expression of intercellular and vascular adhesion molecules promotes the adherence of platelet aggregates, platelet-leucocyte aggregates, and, in severe forms, of erythrocyte aggregates to the endothelium [100,101,102].
(2) During AMI, atherosclerotic material with superimposed thrombotic milieu, originating from the ruptured or eroded plaque, may embolize distally to the microcirculation. Interestingly, the embolized material may aggravate reperfusion beyond the sheer mechanical obstruction mechanism [103]. In fact, the embolization of biologically active material causes patchy micro-infarcts with local inflammatory reactions, aggravating the damage caused by ischemia-reperfusion and intravascular aggregates [104].
(3) In the most severe forms of AMI, the massive swelling of endothelial cells and the consequent interruption of the vascular wall leads to the leakage of circulating blood cells into the interstitial space upon reperfusion, causing IMH [105,106]. IMH represents a negative prognostic factor in patients with AMI together with the angiographic no-reflow phenomenon (NR) [107].

## 3. Therapeutic Strategies against Post-STEMI LVR

The post-STEMI LVR is a reversible phenomenon that can be prevented by early revascularization and dedicated pharmacological therapy [108,109,110]. In particular, anti-platelet agents decrease the risk of stent thrombosis and NR, thereby significantly reducing IS and the incidence of post-AMI LVR. β-blockers, inhibitors of renin-angiotensin-aldosterone system (RAAS), mineralocorticoid receptor antagonists (MRAs), and statins have also been demonstrated to interfere with the development of post-STEMI LVR through direct inhibition of pro-apoptotic and inflammatory pathways.

Further drugs, commonly used for other clinical targets, are currently under investigation for their potential protective role against myocardial cell death following MIRI. These include the selective angiotensin receptor neprilysin inhibitor (ARNI) sacubitril/valsartan (SAC/VAL), proprotein convertase subtilisin/kexin type 9 (PCSK9) inhibitors, novel molecules acting on glucose metabolism, and coronary vasodilators.

Moreover, there are several promising compounds, such as the selective matrix-MMPs inhibitors, interleukin-1 receptor antagonists, anti-inflammatory treatments, selective antioxidant therapy, innate immunity-Toll-like receptors, and nitric oxide-cGMP signaling modulators, that can potentially reduce the risk of post-STEMI LVR onset and are being tested in pre-clinical studies.

In addition to pharmacological therapy, an intriguing research field is represented by mechanical strategies for cardioprotection. Thrombus aspiration (TA) failed to demonstrate an outcome improvement in major randomized clinical trials [111,112,113], but it proved to be useful in particular conditions [114]. The usefulness of ischemic conditioning protocols has not yet been clarified.

Stem cells transfer may represent a very interesting tool for the future, however clinical data about this issue are still sparse.

**Figure 2:**
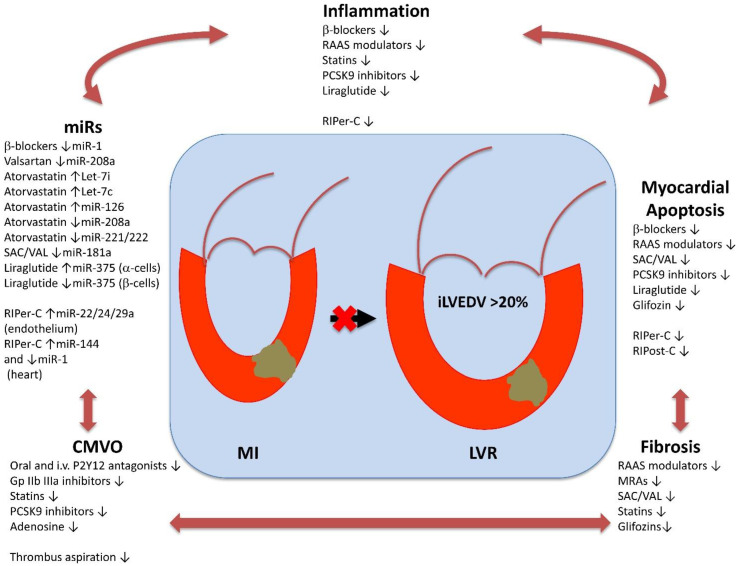
Principal effects of pharmacological therapies and mechanical interventions on pathophysiological determinants of post-STEMI LVR. LVR: left ventricular remodeling; STEMI: ST elevation myocardial infarction; RAAS: renin angiotensin aldosterone system; SAC/VAL: sacubitril/valsartan; PCSK9: proprotein convertase subtilisin/kexin type 9; Gp IIb IIIa; glycoprotein IIb IIIa; RIPer-C: remote ischemic perconditioning; RIPost-C: remote ischemic post-conditioning; miRs: micro-RNAs. ↑ indicates an increase; ↓ indicates a decrease.

In this section, we will discuss conventional and more interesting prospective pharmacological options against post-STEMI LVR, and we will analyze the role of mechanical interventions, such as TA and ischemic conditioning, in MIRI prevention (Figure 2).

### 3.1. Conventional Pharmacological Options

#### 3.1.1. Anti-Platelet Therapy

Dual anti-platelet therapy (DAPT), including acetylsalicylic acid (ASA) plus an oral P2Y12 receptor antagonist, is mandatory in STEMI treatment [115]. Activated platelets specifically infiltrate the ischemic-reperfused myocardium and contribute to MIRI through the formation of micro-thrombi, enhanced platelet-leucocyte aggregation, and the release of potent vasoconstrictor and pro-inflammatory molecules [116].

Anti-platelet agents are not equal, and the choice of a specific oral P2Y12 receptor antagonist should be driven by the pharmacokinetics, pharmacodynamics, and bleeding risk of patient. In STEMI, prasugrel and ticagrelor showed a faster and more effective anti-platelet power leading to a better outcome compared to clopidogrel, but also to an increased incidence of bleeding [7,117,118,119]. No significant statistical difference was detected between prasugrel or ticagrelor in the short- and long-term outcome of STEMI patients [120,121]. However, there is some evidence suggesting the predominant role of the latter in LVR prevention. In fact, it has been demonstrated that ticagrelor increases Ade plasma concentration in patients affected by AMI [122] and exerts, beyond its antiplatelet efficacy, cardioprotective effects by reducing necrotic injury and edema formation via Ade-dependent mechanisms in pig heart [123]. Consistently, in the REDUCE-MVI trial (Reducing Micro Vascular Dysfunction in Acute Myocardial Infarction by Ticagrelor), endothelial function improved over time (1-year follow-up) in ticagrelor patients, while it did not change in the prasugrel group [124].

The timing of anti-platelet therapy is also important and may significantly affect the LVR onset in STEMI patients. Early intravenous ASA administration is always strongly recommended because of its rapid and effective anti-platelet power [115,125]. Conversely, in recent years, the indiscriminate pretreatment strategy (administration of oral P2Y12 antagonists before to know coronary anatomy) has been questioned. In fact, the progressive restriction of “ischemic time” due to rapid performance of pPCI (<1 h) markedly increased the risk of not achieving an effective platelet inhibition at the time of infarct-related artery recanalization, because delayed times of P2Y12 receptor oral antagonists activation during STEMI [126,127]. In this case, a preloading strategy with an intravenous P2Y12 antagonist at fast kinetic activation (cangrelor) or with a glycoprotein IIb IIIa inhibitor (tirofiban) seems to be more suitable to achieve early, powerful platelet inhibition. Interestingly, cangrelor allows a reversible anti-platelet effect with a low bleeding risk and transition to ticagrelor may be started during infusion, thereby minimizing the risk of a newly increased platelet reactivity respect to clopidogrel and prasugrel [128,129]. Furthermore, early administration of cangrelor in STEMI patients was associated with more effective platelet inhibition during pPCI and significantly lowered the deleterious inflammatory response compared to standard anti-platelet therapy [130]. On the other hand, preloading with a glycoprotein IIb IIIa inhibitor determines a non-reversible anti-platelet effect with a higher bleeding risk [131]. Therefore, international guidelines recommend to only use these drugs as a “bail-out” [115]. Nevertheless, a strategy including preloading with tirofiban followed by transition to prasugrel has recently showed a faster inhibition of platelet reactivity compared to cangrelor and prasugrel pretreatment [132]. The clinical potential benefits of this drug association are actually still being tested in the FABOLUS FASTER trial [133].

#### 3.1.2. β-Adrenergic Blockers

Experimental studies have shown the distinctly different and opposite functional roles of β-adrenergic receptor (βAR) 1 and 2 subtypes in regulating cardiac structure and function. In particular, a cardiac protective role of β2-AR signaling has been demonstrated to improve cardiac function and myocyte viability [134,135]; whereas β1-ARs mediate a PKA-independent, calcio-calmodulin kinase II (CaMKII)-dependent, apoptotic and maladaptive remodeling signaling in the heart [136,137]. Metoprolol, a β1-AR selective antagonist, is able to protect against T-tubule remodeling in an experimental model of myocardial infarction [138]. Moreover, early metoprolol administration during ischemia attenuates IS progression and reduces the incidence of primary ventricular fibrillation [139]. In this regard, one of the proposed mechanisms may be represented by miR-1 expression down-regulation leading to Cx43 up-regulation [140].

In the clinical METOCARD-CNIC (Effect of Metoprolol in Cardioprotection During an Acute Myocardial Infarction) trial, early intravenous metoprolol (15 mg) before reperfusion reduced IS and increased left ventricular ejection fraction (LVEF), in STEMI patients with anterior Killip class II or less undergoing pPCI [141]. In a post hoc analysis of this study, it has also been demonstrated that the sooner the intravenous metoprolol administration, the smaller the IS and the higher the LVEF [142]. Conversely, the EARLY BAMI trial (Early Beta Blocker Administration Before Reperfusion Primary PCI in Patients With ST-Elevation Myocardial Infarction) failed to report a reduction in IS at one month with intravenous metoprolol (2 × 5 mg) administered just before pPCI in patients with STEMI presenting within 12 h of symptom onset [143].

Thus, the most recent European guidelines suggest the administration of intravenous metoprolol at STEMI diagnosis in the presence of hemodynamic stability and in the absence of contra-indications (such as marked hypotension and bradycardia, or atrio-ventricular blocks) [115].

#### 3.1.3. RAAS Antagonists

The RAAS has been intensively studied in the development of LVR following AMI.

In the heart, angiotensin II has multiple direct cytotoxic effects on cardiomyocytes: inducing apoptosis, promoting cell hypertrophy, and stimulating myocardial fibrosis via angiotensin II type 1 receptor (AT-1R). AT-1R exerts most of the physiological effects of angiotensin II: vasoconstriction, increased aldosterone release, and potentiation of sympathetic activity. Angiotensin II receptor 2 (AT-2R) is thought to cause the opposite effects of AT-1R. A higher density of AT-1 receptors, as detected on blood platelets, may confer a greater risk of undergoing LVR for up to six months after an AMI [144,145]. Moreover, angiotensin converting enzyme (ACE) is implied in the degradation of bradykinin (Bk), which is a biomolecule that plays a protective role in endothelial cells [146] and induces reparative processes in the myocardium [147] against hypoxic injury. High plasmatic levels of Bk have been detected in AMI survivors, and this finding has been related to the lower kininase activity in the lung or in the circulating blood compared to non-survivors [148].

Blocking ACE activity may play a dual positive role, because it antagonizes angiotensin II production and Bk degradation, thereby promoting cardioprotection against MIRI. In patients undergoing pPCI, the injection of ACE-inhibitor enalaprilat in the infarct-related artery has been shown to reduce the adhesion of inflammatory cells and improve epicardial flow [149] through a significant increase of Bk in pulmonary arterial blood [150]. The intra-coronary administration of ACE-inhibitors has not yet been introduced in clinical practice. However, it is widely accepted that early treatment with oral ACE-inhibitors in STEMI is safe, well tolerated, and associated with a significant reduction in 30-day mortality, especially in the first week after acute ischemic event [151,152] and in patients with reduced LVEF (<40%) or who have developed acute HF [151,153,154,155,156].

The selective blocking of AT-1R also protects against post-AMI LVR through the indirect stimulation of AT-2R in animal models. In fact, under AT-1R blockade with valsartan, the AT-2R-deficient mice revealed no remodeling protection from valsartan [157]. Thus, in all non-tolerant patients to ACE-inhibitors, an angiotensin receptor type 1 blocker (ARB) should be administered [115,158].

More recent agents impeding the RAAS at the earliest point, such as the direct renin inhibitor aliskiren, have been shown to reduce LVR with decreased apoptosis and myocardial scarring in murine infarcted-heart models [159]. However, adding aliskiren to the standard therapy, including an inhibitor of the RAAS, in high-risk post-MI patients did not result in further attenuation of LVR, and was associated with more adverse effects [160]. These findings do not suggest that dual RAAS blockade with aliskiren would provide additional benefits to these high-risk post-MI patients.

Aldosterone is an important mineralocorticoid hormone, which regulates plasma sodium and potassium concentrations, and, through feedback mechanisms, can activate the RAAS pathway. Aldosterone plays a role in LVR by stimulating cardiac collagen synthesis, including collagen type I and type III [161]. The effect of selective mineralocorticoid receptor agonists (MRAs) has been studied in post-AMI patients with HF. Eplerenone has been shown to reduce morbidity and mortality in these patients [162]. Two more recent trials with eplerenone [163] and with single potassium canrenoate intravenous bolus followed by spironolactone [164], respectively, demonstrated the benefit of an early MRA administration in STEMI without HF compared to placebo. Early administration of MRAs is recommended in patients with reduced LVEF (<40%) or who developed an acute HF after STEMI [115,162,165,166,167].

Actually, none of the microRNAs previously linked to cardiac fibrosis (mir-1, mir-21, mir-29a, mir-29b, mir-101, mir-122, mir-133a) predicted an antifibrotic response to eplerenone antagonism [168]. Conversely, it has been demonstrated that treatment with valsartan can decrease myocardial fibrosis through attenuating miR-208a and endoglin expression [42].

#### 3.1.4. Statins

A recent analysis has demonstrated that LDL-cholesterol (LDL-C) and triglycerides are associated with adverse changes in cardiac structure and function [169]. In fact, the achievement of low LDL-C levels with statin therapy has been demonstrated to reduce the incidence of post-STEMI LVR [170].

It is also well known that the reduction in CV events by statins is significantly greater than that resulting from the reduction in lipid levels [171]. Data from randomized clinical trials and meta-analyses indicate that the early use of a high-dosage statin therapy after STEMI is associated with rapid and prolonged clinical benefits [172,173]. In fact, the administration of an atorvastatin loading dose before pPCI was associated with a decreased CMVO incidence [174]. At the same time, ongoing statin therapy at the time of STEMI was associated with a lower rate of CMVO, a better functional recovery of myocardial function after six months of follow-up [175], and a reduced IS [176] when compared with patients not on statins. A post hoc analysis from the SECURE-PCI trial (Statins Evaluation in Coronary Procedures and Revascularization) showed that the subgroup of pPCI patients had a nearly 50% reduction in 30-day major adverse cardiac events (MACE) with high-dose atorvastatin (administered prior and 24 h after pPCI) compared with placebo [177]. Similarly, a high loading-dose of rosuvastatin (20 mg) before pPCI caused a decrease in MACE [178].

These results depend on the pleiotropic actions of statins, including anti-platelet and anti-coagulant power, as well as anti-inflammatory and anti-fibrosis effect, and improved endothelial function [179,180].

Statins can lower the level of LDL-C in the plasma membrane of platelets, thereby reducing their reactivity [181]. Moreover, randomized studies have shown a significant decrease in the plasma concentration of factor VIII (FVIII), which is related to a major incidence of AMI relapses [182], in the group of patients taking high doses of statins [183].

The anti-inflammatory effect of atorvastatin can arise from upregulation of miR Let-7i expression in monocytes, thereby down-regulating the Toll-like receptor 4 (TLR4) signaling pathway that is implied in the activation of atherosclerotic plaque [184]. Furthermore, atorvastatin prevented oxidized-LDL from inducing miR Let-7c in dendritic cells; hence, the plaque T cell proliferation and following rupture were abrogated [185,186]. Moreover, atorvastatin upregulated miR-126 expression and suppressed VCAM-1 protein expression, that is required the acceleration of the plaque formation [187].

Treatment with atorvastatin can also decrease myocardial fibrosis through attenuating miR-208a and endoglin expression in experimental AMI [42]. This effect prevents the major extension of myocardial scars, thereby reducing the risk of LVR. In addition, atorvastatin decreases miRs-221/222 expression, thereby enhancing angiogenesis through modulation of endothelial nitric oxide synthase (eNOS) in patients with CV disease [188].

Definitively, the statin-induced miR network would affect the integrin-signaling pathway in vascular endothelial cells and platelets, while altering differentiation in monocytes, thus leading to atherosclerotic plaque stability [189]. European guidelines recommend beginning high-dosage statin therapy in all naïve patients affected by STEMI and without contraindications, independently of LDL-C values, within four days of STEMI [190]. A low-dosage statin therapy should be limited to patients with a well-defined increased risk of collateral effects, such as elderly, those with altered renal and hepatic functions, or recognized intolerance [190].

#### 3.1.5. Ezetimibe

In the IMPROVE-IT (Improved Reduction of Outcomes: Vytorin Efficacy International Trial) trial, ezetimibe added to simvastatin allowed the improvement of the outcome of post-STEMI patients [191]. This benefit was maintained in all subgroups of patients [192] and determined a reduction in total cardiovascular events [193], ictus cerebri [194], and re-hospitalizations. In particular, patients at higher thrombotic risk mostly benefited from ezetimibe addition [195].

### 3.2. Prospective Pharmacological Options

#### 3.2.1. Neprilysin Inhibition

An early event in STEMI is the marked release of natriuretic peptides (NPs) from the myocardium [196], followed by a 10-fold increase in Bk plasmatic levels within 48 h [148]. Both these molecules exert a protective effect against MIRI [146].

Neprilysin (NEP) is the most important aminopeptidase required for degradation of NPs [197], but it is also implied in Bk catabolism. In particular, during STEMI, NEP plays a more relevant role in Bk degradation compared to ACE, because ACE activity is dominant at lower Bk levels (physiologic conditions), whereas NEP activity is dominant at higher Bk concentrations (AMI) [198]. Furthermore, NEP is required for enzymatic inactivation of other cardioprotective peptides, such as apelin, substance P (SP), and adrenomedullin (ADM) [146,197]. Thus, it is conceivable that NEP inhibition could determine more benefits than ACE-inhibitors in MIRI antagonism.

Given these findings, SAC/VAL (LCZ696) could be an attractive candidate for cardioprotection against MIRI and following LVR. SAC/VAL is a first-in-class approved ARNI that simultaneously provides NEP inhibition and AT-1Rs blockade. Concomitant NEP and AT-1Rs antagonism may increase levels of peptides leading to activation of several pro-survival pathways (NPs, Bk, apelin, SP, ADM) and inhibition of myocardial fibrosis [199,200]. In this regard, it has recently been demonstrated that treatment with SAC/VAL resulted in the increased production of exosomes containing regulatory small molecules, such as miRs, by induced pluripotent stem cell-derived cardiomyocytes. Sequencing of these exosomes exhibited down-regulation of miR-181a resulting in the attenuation of myocardial fibrosis and hypertrophy, thereby restoring an injured rodent heart after AMI [201]. Furthermore, SAC/VAL may inhibit pro-apoptotic mechanisms mediated by acute angiotensin II increase. Interestingly, patients who began taking SAC/VAL for acute HF in the hospital had a lower hazard for the composite outcome compared with patients that initiated enalapril in the hospital and then had a delayed initiation of SAC/VAL [202].

In fact, there are no data to support SAC/VAL administration in acute STEMI. It is hoped that encouraging evidence may come from the results of the ongoing PARADISE MI study (NCT02924727).

#### 3.2.2. PCSK9 Inhibitors

PCSK9 can enhance the degradation of LDL-receptor (LDL-r) and its closest structural family members, thereby increasing vascular inflammation and affecting ECM homeostasis [203]. PCSK9 may also contribute to the degradation of other receptors, including CD36, which is a regulator of platelet aggregation [204]. Circulating PCSK9 levels are spontaneously augmented in the case of AMI [205] and are associated with higher platelet reactivity and the risk for atherothrombotic events during the 1-year follow-up of patients with acute coronary syndrome [206]. Interestingly, PCSK9 and autophagy were significantly upregulated in cardiomyocytes exposed to hypoxia and they were showed to extend the infarct area in mouse hearts subjected to left coronary artery occlusion [207].

The monoclonal antibodies alirocumab and evolocumab are selective inhibitors of PCSK9. They were showed to improve the outcomes of patients with a previous AMI [208,209]. In particular, the closer the PCSK9 inhibitor’s administration was to the AMI, the greater the benefit [210,211]. These results may be attributed to their very rapid LDL-C lowering effect [212,213,214,215], mediated by higher LDL-r availability on the epatocyte surface, increased high-density lipoprotein (HDL) concentrations, and reduced lipoprotein-a (Lp-a) levels [216,217].

However, a previous meta-analysis reported that the reduction in CV events is greater in patients using PCSK9 inhibitors compared to subjects using other lipid-lowering therapies with the same level of LDL-C reduction [218]. The reasons for this benefit from PCSK9 inhibition during AMI seem to go beyond lipid metabolism and may be due to pleiotropic effects. In fact, PCSK9 inhibitors play an anti-inflammatory effect in atherosclerotic plaque that is complementary to statin action. High-dosage atorvastatin (80 mg) increases PCSK9 plasmatic levels [219]. PCSK9 upregulation by statin therapy and AMI exacerbates the inflammatory state of stable atherosclerotic plaque, thereby allowing its translation to a vulnerable plaque that is more prone to rupture and thrombosis [220].

Furthermore, PCSK9 inhibitors may exert a cardioprotective effect through the antagonism of thrombosis. In fact, because of their ability to reduce lectin-like oxidized low-density lipoprotein receptor-1 (LOX-1) expression on the surface of platelets [221] and transport of Lp-a by lipid-peroxide-modified phospholipids [222], PCSK9 inhibitors may contribute to the reduction in platelet activity. Moreover, PCSK9 inhibitors antagonize the interaction between PCSK9 protein and CD36 receptor on platelet surface, thereby affecting CMVO, the risk of NR, and the following IS extension [223]. Consistently, lower circulating levels of PCSK9 were found to be inversely associated with LVEF at six months since the STEMI event [224].

These findings seem to suggest an early administration of PCSK9 inhibitors in AMI. In fact, European guidelines recommend treatment with PCSK9 antagonists in patients affected by AMI and who have not reached LDL-C therapeutic target after 4–6 weeks of maximum tolerated statin plus ezetimibe therapy [190]. In patients still undergoing active statin plus ezetimibe treatment, PCSK9 inhibitors would be administered during hospitalization [190]. Thus far, there are no data about the immediate use of PCSK9 antagonists in statin-naïve ischemic patients.

#### 3.2.3. Novel Drugs Modulating Glucose Metabolism

Stress-induced hyperglycemia (SIH) at hospital admission for AMI is a very common condition and is associated with poor outcomes, especially in patients without known diabetes [225,226,227,228]. SIH in the context of an AMI, compared to that in known diabetes, represents an epiphenomenon of neuro-humoral alterations [229]. However, the extension of IS correlated with glucose levels at the time of presentation, with greater infarct areas observed in non-diabetic than in diabetic patients presenting with similar blood glucose levels [230]. The association between hyperglycemia upon hospital admission and IS in STEMI patients is a consequence of a larger myocardial area at risk [231]. Thus, SIH may be not only considered as a marker of endocrine alterations occurring during AMI, but also as a mediator of MIRI.

Interestingly, insulin treatment in AMI patients was not correlated with a reduction in mortality [232,233]. This is probably due to the fact that SIH directly impairs insulin signaling [234]. Nevertheless, a tighter glycaemic control leads to a better prognosis [235,236,237], although hyperglycemia does not influence the effect of the reperfusion treatment [231]. Therefore, drugs both accounting for cell survival and modulating glucose metabolism might be needed for cardioprotection against MIRI. In this regard, molecules such as glucagon-like peptide 1 receptor agonists (GLP-1 RAs), dipeptidyl peptidase-4 inhibitors (DPP-4Is), and sodium-glucose co-transporter 2 inhibitors (SGLT-2Is), commonly used for diabetes treatment, showed promising results.

GLP-1 RAs exert multiple glucose-regulatory actions. In fact, they decelerate gastric emptying, stimulate insulin secretion (β-cells), and suppress glucagon release (α-cells) [238]. The improved salvation of myocardium at risk for necrosis with intravenous [239] and a reduced IS with subcutaneous GLP-1 RA exenatide has been demonstrated [240]. Liraglutide treatment reduced the resulting necrotic area [241] and improved LVEF after pPCI for STEMI [242] and non-STEMI [243]. The mechanisms of cardioprotection mediated by GLP-1 RAs may be attributed to the scavenging of oxidative stress products, an increase in the concentrations of antioxidant defense enzymes, and the inhibition of cardiomyocyte apoptosis [244]. Moreover, interestingly, liraglutide has been shown to induce cell apoptosis in pancreatic α-cells through the increase of miR-375 and improve cell viability in pancreatic β-cells through the down-regulation of miR-375 [245]. Thus, we may speculate that the cardioprotective effect of liraglutide is also mediated by modulation of glucose metabolism.

DPP-4Is reduce degradation of GLP-1, thereby resembling the action of GLP-1 RAs [238]. Nevertheless, the results of large-scale clinical trials on cardioprotection with DPP-4Is were neutral [246].

SGLT-2Is, also known as glifozins, are a novel class of antidiabetic drug that reduce the reabsorption of glucose and sodium from the proximal convoluted tubules, resulting in glycosuria and natriuresis properties [247]. The EMPA-HEART trial has demonstrated that empagliflozin decreased LV mass after six months in diabetic patients and either previous coronary revascularization or history of AMI, thus suggesting that SGLT2is may lead to an improvement in LVR after AMI [248]. Consistently, an analysis from the DECLARE-TIMI 58 trial showed lower rates of MACE with dapagliflozin in patients with previous AMI [249]. This benefit seemed to be higher the closer it was to the acute event.

Finally, whereas controversial data are available about the potential benefit of GLP-1 RAs and DDP-4Is in AMI patients with previous or developing HF [250,251,252,253,254,255], dapagliflozin consistently reduced the composite endpoint of CV death or hospitalizations for HF in this population [249]. Therefore, SGLT-2Is should be preferred to GLP-1 RAs and DPP-4Is in HF patients with previous AMI. To further address this result, a phase 3b trial has very recently been proposed [256].

#### 3.2.4. Coronary Vasodilators

Intra-coronary vasodilators were tested as pharmacological treatments of acute NR. Among these, Ade, an endogenous nucleoside characterized by a short half-life (<2 s) [257] was the most effective at reducing NR incidence by inducing relaxation of coronary micro-vascular circulation [258,259]. Ade also exhibits anti-inflammatory properties against neutrophils and inhibits platelet aggregation [260]. Moreover, Ade mimics ischemic preconditioning, limiting reperfusion injury, exhibiting antiapoptotic effects, and perhaps stimulating angiogenesis [260]. However, data from clinical studies with Ade are controversial.

In AMISTAD-1 trial, Ade within 6 h of STEMI onset reduced IS compared to placebo in patients undergoing fibrinolysis [261]. Conversely, the more recent AMISTAD-2 study demonstrated that high-dose Ade did not improve short-term clinical outcomes after anterior STEMI and undergoing fibrinolysis or pPCI, although there was a significant reduction in necrotic area extension [262]. Nevertheless, in a follow-up analysis of the AMISTAD-2 trial, patients who received an early reperfusion in addition to Ade showed a significant reduction in HF incidence and mortality [263]. In the REOPEN-AMI trial, Ade was compared to sodium nitroprussiate and placebo after TA. In this case, Ade showed a positive effect on ST-resolution [264]. In the more recent REFLO-STEMI study, Ade-treated patients who had undergone pPCI presented a slight increase in 30 day- and six month- MACE [265].

Thus, high-dosage Ade seems to be contra-indicated and low-dosage also needs to be reconsidered in STEMI patients.

### 3.3. Mechanical Interventions

#### 3.3.1. Thrombus Aspiration

Routine TA during pPCI for STEMI did not reduce long-term clinical outcomes and may even be associated with an increase in stroke [111,112,113]. As a result, TA is no longer recommended as a routine strategy in STEMI patients [115].

However, the protective role of TA in LVR has been shown in several smaller clinical trials. For example, it has been indicated that TA could protect against six-month LVR remodeling in a retrospective analysis of 109 STEMI patients [266]. Similarly, a randomized controlled trial found that TA showed a smaller iLVEDV than the conventional group did after a six-month follow-up [267]. In addition, both the EXPIRA (Thrombectomy With Export Catheter in Infarct-Related Artery During Primary Percutaneous Coronary Intervention) [268] and MUSTELA (Multidevice Thrombectomy in Acute ST-Segment Elevation Acute Myocardial Infarction) [269] trials demonstrated the protective effect of TA on CMVO extent. A further randomized clinical study showed that manual TA in the setting of pPCI improves myocardial tissue-level perfusion as well as LV functional recovery and LVR [270]. More recently, we demonstrated that TA during pPCI for STEMI reduces clinical outcomes in hyperglycemic patients [114].

Therefore, the usefulness of TA needs to be further studied in more selective settings and cases of STEMI.

#### 3.3.2. Remote Ischemic Perconditioning

Remote ischemic perconditioning (RIPer-C) is defined as a phenomenon in which brief cycles of ischemia and reperfusion, which have been applied to an organ or tissue far from the heart before or during reperfusion, reduce myocardial IS. The molecules triggering the cardioprotective mechanism of RIPer-C have not been thoroughly identified. However, several experimental and clinical studies have indicated that nitric oxide (NO) [271], opioids [272], Ade [273], Bk [146,274], and cytokines [275] are involved. Furthermore, extracellular vesicles, which are lipid bilayer-coated particles secreted by most cell types into the extracellular space and subsequently into the circulation, have been identified as potential carriers of cardioprotective signals of RIPer-C [276,277]. Extracellular vesicles are widely enriched with different miRs, whose stability is further improved by this system of transport in the blood. Preconditioned endothelial cells represent an important source of microvesicles that are able to evoke higher protection against ischemia-reperfusion injury in cardiomyocytes [278]. More interestingly, it has been shown that transfusion of microvesicles isolated from rats immediately, but not 6 h after a hind limb ischemia-induced RIPer-C, into recipient rats exposed to heart ischemia-reperfusion injury resulted in IS reduction and improved functional recovery of the heart [279,280]. Thus, it is conceivable that RIPer-C may represent an interesting ‘bridge’ to classic pharmacological therapy.

Several miRs seem to be intimately involved in the cardioprotection evoked by RIPer-C. While certain miRs (miR-22, miR-29a, mir-24) are transported in RIPer-C-released extracellular vesicles in order to mediate the cardioprotective signal by humoral transport from conditioned organ to the heart [281], the expression of other miRs (miR-1, miR-144) is regulated by RIPer-C within the heart tissue, thereby suggesting that they are post-receptor mediators of this phenomenon in the heart [282].

For these reasons, RIPer-C is now considered a useful tool for cardiac protection, although some technical aspects (such as timing and site of application, as well as the precise number of ischemia-reperfusion cycles in order to reach major benefit in clinical outcome) have not been yet clarified [283,284,285], and the results from clinical studies are still controversial.

In humans, the cardioprotective RIPer-C stimulus can be applied using serial inflations and deflations of a pneumatic cuff placed on the upper arm or thigh to induce brief cycles of ischemia and reperfusion [286]. In most clinical STEMI studies, RIPer-C has increased myocardial salvage and reduced myocardial IS by 20–30% [285,287,288,289,290,291]. Nevertheless, only in two follow-up studies has the myocardial IS reduction by RIPer-C correlated with an improvement in clinical outcomes in patients with STEMI undergoing pPCI [292,293]. In particular, a follow-up of participants in the initial CONDI-1 (Remote Ischaemic Conditioning Before Hospital Admission, as a Complement to Angioplasty, and Effect on Myocardial Salvage in Patients with Acute Myocardial Infarction) trial [283] showed that increased myocardial salvage with RIPer-C was associated with reduced frequencies of MACE compared with the control group [292]. Consistently, follow-up of participants in the LIPSIA CONDITIONING (Cardioprotection by Combined Intrahospital Remote Ischaemic Perconditioning and Postconditioning in ST-Elevation Myocardial Infarction) trial revealed that MACE (cardiac death, reinfarction, and new congestive HF) was reduced in the group that received combined RIC and ischemic post-conditioning (Post-C) compared with the control group (patients who received pPCI alone) or patients receiving ischemic Post-C with pPCI [293]. Unfortunately, these results were not confirmed by the largest, appropriately powered, prospective, CONDI-2/ERIC-PPCI (Effect of Remote Ischaemic Conditioning on Clinical Outcomes in STEMI Patients Undergoing PPCI) trial. In fact, although RIPer-C reduced platelet reactivity in the first 48 h post-STEMI [294], no clinically relevant beneficial effect on clinical outcomes (cardiac death or hospitalization for HF) was found after 12 months in patients with STEMI when compared with pPCI alone [295].

The reasons for the difficulty in translating positive results achieved in experimental models into clinical benefits are not completely known, but probably derive from different causes. First of all, most of RIPer-C studies presents the reduction in myocardial IS as the primary endpoint. Although myocardial IS represents a well-defined, independent determinant of clinical outcomes post-pPCI in patients with STEMI [27], it is unclear whether a reduction in myocardial IS by a cardioprotective intervention applied as an adjunct to pPCI can be translated into improved clinical outcomes. Conversely, the prevention of LVR is a well-defined marker for improved prognosis in STEMI patients [13], but this parameter was not analyzed in the CONDI-2/ERIC-PPCI trial. As a support to this hypothesis, the RIC-STEMI trial showed no reduction in myocardial IS, but still found a positive effect on post-STEMI LVR, leading to fewer cardiac deaths and hospitalizations for HF after a median follow-up time of 2.1 years [284].

Furthermore, experimental data have shown that age and presence of comorbidities, including diabetes, dyslipidemia, and hypertension might attenuate the cardioprotective effects of RIPer-C [296]. Contemporarily, comedications might affect the cardioprotective efficacy of RIPer-C. In a recent experimental study, it has been demonstrated that a combined background therapy including an opioid agonist (enkephalin), heparin, and a platelet-inhibitor (ticagrelor) were protective by themselves, reducing IS, whereas RIPer-C did not add any further protection [297]. Most of the drugs commonly used to reduce myocardial injury in acute STEMI (β-blockers, RAAS modulators, statins, platelet antagonists), as well as those being tested for cardioprotection (ARNI, GLP-1 RAs, DPP-4Is, SGLT-2Is, and PCSK9 inhibitors) interact with pathophysiological mechanisms triggered by RIPer-C, thereby confounding the cardioprotective effect of this intervention.

Finally, the timing of the RIPer-C protocol application in relation to reperfusion by pPCI might be very important. Previous clinical studies have shown that RIPer-C is effective at reducing myocardial IS, especially when administered before pPCI (either in transit to the pPCI center or on arrival at the hospital) [283,285].

#### 3.3.3. Classic and Remote Post-Conditioning

Classic ischemic Post-C, consisting of brief repeated cycles of PCI balloon inflation-deflation in the culprit coronary artery after reperfusion onset, gave controversial results in clinical trials and its usefulness has been strongly questioned in recent years. In fact, the RIPOST-MI (Remote Ischemic POSTconditioning in Myocardial Infarction) study showed that addition of post-C to RIPer-C did not lead to a further decrease in IS compared to RIPer-C alone [291]. Similarly, in the LIPSIA CONDITIONING trial, post-C alone failed to improve myocardial salvage and CMVO, whereas combined post-C with RIPer-C improved myocardial salvage [287], which translated to a reduced rate of MACE and new congestive HF after STEMI [293]. Consistently, the DANAMI-3 iPOST (The Third Danish Study of Optimal Acute Treatment of Patients With ST Elevation Myocardial Infarction–Ischemic Postconditioning) trial demonstrated that routine Post-C during pPCI failed to reduce the composite outcome of death from any cause and hospitalization for HF in patients with STEMI and TIMI grade 0–1 flow at arrival [298]. Conversely, in an NHLBI sponsored randomized trial, Post-C was associated with improved LVR at one year follow-up in subjects with CMVO, although no early benefit on IS, myocardial salvage index, and LV function was observed compared with routine pPCI [299]. However, the study population was very small and Post-C was applied before stent implantation (within 1 min since reperfusion). Therefore, the benefit from Post-C might be overestimated because of the relatively limited sample size, the absence of direct stenting, and the much lower TA use (42% in control group and 23% in post-C group) compared to previous trials.

Interestingly, when Post-C was applied by cuff inflation/deflation of the inferior limb (remote post-C or RIPost-C) and TA was strongly encouraged in patients with anterior STEMI, a reduced enzymatic IS with an improvement of edema volume and ST-segment resolution >50% were observed [300]. In this regard, we may hypothesize that RIPost-C, while maintaining protective effects, reduces the risk of thrombus embolization and of consequential CMVO during pPCI compared to classic Post-C.

## 4. Reasoned Multitarget Therapeutic Strategy against Post-STEMI LVR

**Figure 3:**
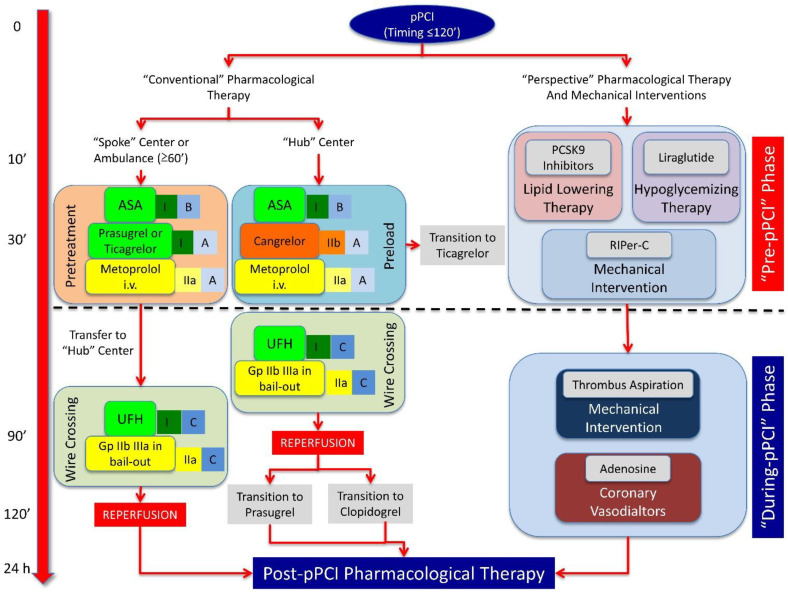
Scheme for a reasoned multitarget therapeutic strategy against post-STEMI LVR, in the ‘pre-PCI’ and ‘during-pPCI’ phases. On the left side of the picture, the timing of conventional pharmacological therapy for STEMI patients is described, in the ‘pre-PCI’ and ‘during-pPCI’ phases, according to the European Society of Cardiology (ESC) guidelines. Each pharmacological indication is identified with its own class of recommendation and level of evidence. On the right side of the picture, perspective pharmacological therapies and mechanical interventions in order to further improve protection against post-STEMI LVR according to precise timing are listed. PCSK9 inhibitors, liraglutide, and RIPer-C should be administered in the ‘pre-PCI’ phase, whereas thrombus aspiration (especially in patients with high glycemic values) and adenosine (in case of no-reflow phenomenon) should be used in the ‘during-pPCI’ phase. STEMI: ST elevation myocardial infarction; pPCI: primary percutaneous intervention; LVR: left ventricular remodeling; PCSK9: proprotein convertase subtilisin/kexin type 9; RIPer-C: remote ischemic perconditioning; ASA: acetylsalicylic acid; UFH: unfractioned heparin; Gp IIb IIIa: glycoprotein IIb IIIa.

**Figure 4:**
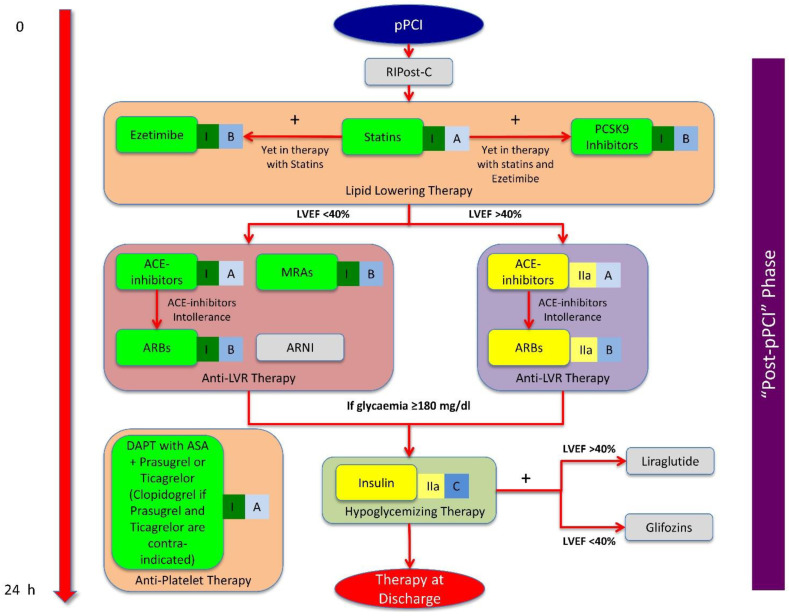
Scheme for a reasoned multitarget therapeutic strategy against post-STEMI LVR in the ‘post-pPCI’ phase. In this picture, the timing of conventional pharmacological therapy for STEMI patients, in the ‘post-pPCI’ phase is described, according to the European Society of Cardiology (ESC) guidelines. Each pharmacological indication is identified with its own class of recommendation and level of evidence. In the gray rectangles, the perspective pharmacological therapies (ARNI, liraglutide, glifozins) and mechanical interventions (RIPost-C) in order to further improve protection against post-STEMI LVR according to precise timing are identified. STEMI: ST elevation myocardial infarction; pPCI: primary percutaneous intervention; LVR: left ventricular remodeling; ARNI: angiotensin receptor neprilysin inhibitor; RIPost-C: remote ischemic post-conditioning; PCSK9: proprotein convertase subtilisin/kexin type 9; ARBs: angiotensin receptor blockers; MRAs: mineralocorticoid receptor antagonists; DAPT: dual anti-platelet therapy; ASA: acetylsalicylic acid; LVEF: left ventricular ejection fraction.

The prevention of MIRI and following post-STEMI LVR is a time-dependent phenomenon and may be improved through an integrated (pharmacological and mechanical) multitarget therapeutic strategy, where the correct sequence of application of protective interventions makes the difference. In this context, we can distinguish three distinct phases: (1) “pre-pPCI” phase, (2) “during-pPCI” phase, and (3) “after-pPCI” phase (Figure 3 and Figure 4).

### 4.1. “Pre-pPCI” Phase

This is the most critical phase for the prevention of MIRI and of IS extension, because drugs and mechanical interventions must efficiently trigger pathophysiological protective pathways in the myocardium to fight the lethal effects of reperfusion (Figure 3). The most favorable scenario is represented by a large STEMI (especially anterior), because cardioprotection is most needed and easy to demonstrate when the damage by MIRI is at its greatest. Furthermore, ‘ischemic time’ has great relevance because, with early pPCI, reperfusion per se may be sufficient to salvage the myocardium, and no additional cardioprotection may be required. Thus, the time window where adjunct cardioprotection truly rescues the reperfused myocardium from infarction is relatively small, and is in fact limited to a few hours (<12 h; optimal range 2–6 h) [301]. Pre-infarction angina is frequent in patients with STEMI, and it may confer preexisting protection [302], thus, these subjects must not be included in cardioprotection trials. Bypass history and administration of anesthetic drugs are also exclusion criteria for recruitment of patients in a multitarget cardioprotective strategy trial. In fact, the collateral blood flow provided by a normally working bypass in cardiac non-ischemic regions may guarantee minimal perfusion in the infarcted area, thereby blunting the cardioprotective effect. Similarly, non-volatile anesthesia (propofol) is a confounder in assessing the efficacy of a cardioprotective drug or intervention and could obscure potential cardioprotection [303,304].

The latest European guidelines on STEMI treatment underline the importance of starting antiplatelet therapy early in order to reduce the risk of stent thrombosis and the NR phenomenon. To this end, a ‘pretreatment strategy’ with oral P2Y12 antagonists in spoke centers and in ambulance (when ‘ischemia-to-pPCI’ time is >1 h) or ‘preloading strategy’ with cangrelor in hub centers (when ‘ischemia-to-pPCI’ time is <1 h) is recommended in addition to aspirin [115].

Similarly, intravenous metoprolol, in the presence of hemodynamic stability and the absence of contraindications, must be administrated because of its protective role on LV function [115].

The idea of PCSK9 inhibitor administration, independent of previous statin therapy, in this phase is intriguing. We believe that the addition of PCSK9 inhibitors to statin therapy may increase its protective effect against MIRI, thereby reinforcing the anti-inflammatory and anti-platelet activity of statins. Moreover, a single administration to counteract acute elevation of PCSK9 levels during STEMI is reasonable, limiting chronic therapy with these drugs to patients being treated with the maximum tolerated statin dosage plus ezetimibe who have LDL-C levels over the target limit identified by the European guidelines [190].

Liraglutide must be included in the pharmacological armamentarium of the pre-pPCI phase, because it has been shown to improve LVEF in STEMI patients independently on diabetes status [242]. This drug is obviously contraindicated in the case of patients with or experiencing hypoglycemia or diabetic ketoacidosis.

The results of the CONDI-2/ERIC-PPCI trial reinforce the concept that RIPer-C alone cannot provide an improvement in prognosis in STEMI patients, but it needs to be included in a multitarget strategy. In fact, RIPer-C, through the rapid activation of survival pathways, may blunt MIRI at reperfusion onset while oral and intravenous cardioprotective drugs become active.

### 4.2. ‘During-pPCI’ Phase

The angiographic characteristics and technical aspects of pPCI are important for the success of a multitarget cardioprotective strategy. Patients could have thrombolysis when their myocardial infarction (TIMI) flow is 0–1 at admission and they have non-visible collaterals. In fact, patients who have a TIMI flow >0–1 or collateral flow in the infarcted area have likely already experienced some reperfusion prior to pPCI, and are thus potentially conferred protection by gentle reperfusion [305]. It is obviously impossible to know the status of coronary arteries before coronary angiography. Therefore, the pharmacological and mechanical interventions in the ‘pre-pPCI’ phase must be performed independently with the knowledge of angiographic characteristics, which, however, must be adequately considered in the analysis of clinical results.

There are no definitive data about usefulness of classical post-C in this phase, because positive effects have only been reported when this intervention was applied before stent implantation [299]. Moreover, STEMI patients would ideally undergo direct stenting [306], because further manipulation of the culprit lesion by the post-C balloon inflation/deflation may otherwise cause coronary microembolization [307] and exaggerate myocardial damage [308]. Nevertheless, direct stenting at a TIMI flow of 0, when the coronary anatomy beyond the occlusion is uncertain, is a problem, and therefore not performed in many interventional laboratories. For these reasons, we believe that classical post-C should be avoided, whereas just a single protocol of RIPost-C may be used in the attempt to further extend the protective window provided by RIPer-C against MIRI (Figure 4).

A post-pPCI TIMI flow grade <2 may be a marker of NR, which negatively impacts cardioprotection. Interestingly, in more than 30% of the patients with TIMI 3 flow and myocardial blush grade (MBG) 2–3, ST-segment resolution is incomplete after reperfusion, representing an independent marker of CMVO [309]. NR and CMVO incidence could be reduced by specific pharmacological and mechanical interventions, even if there is no widespread consensus about their routine use in the ‘during-pPCI’ phase (Figure 3). Ade has been widely tested, but results from clinical studies are extremely controversial [261,264,310,311,312]. Similarly, Gp IIb IIIa inhibitors are recommended in ‘bail-out’ because of the related higher bleeding risk [115]. TA may be used in the case of high thrombus burden, especially in patients with hyperglycemia at admission [114].

### 4.3. ‘Post-pPCI’ Phase

The post-pPCI phase is the phase in which the protective effects triggered before pPCI must be perpetuated to prevent LVR (Figure 4).

DAPT is the cornerstone of post-pPCI therapy [115]. β-blockers, statins at maximum tolerated dosage, and ezetimibe must be prescribed to prolong the beneficial effects started in the ‘pre-pPCI’ phase. The initiation of a therapy with RAAS modulators (ACE inhibitors or ARBs) is strongly recommended [115].

Therapy with high-dosage statins and ezetimibe is also mandatory in this phase due to their anti-platelet and anti-coagulant power, as well as their anti-inflammatory and anti-fibrosis effects [179,180].

Liraglutide could be continued, with or without insulin, especially in the case of persistent hyperglycemia and preserved LVEF, for the first seven days [242]. Conversely, in the case of acute reduced LVEF after STEMI, glifozins [313] and ARNI [202] treatment should be chosen.

The infusion of Gp IIb IIIa inhibitors may be prolonged for 12 h in the case of their ‘bail-out’ utilization during pPCI and evidence of a large thrombus burden [115].

### 4.4. Proposal for a Randomized Clinical Trial

Here, we try to propose the design for a new randomized trial based on consistent studies mentioned in this text.

We believe that the choice of an adequate study population will allow us to more easily demonstrate the efficiency of cardioprotective pharmacological and mechanical interventions. In particular, recruited patients will have to present a large STEMI (LVEF ≤ 40%; preferably anterior STEMI), with an ‘ischemic time’ between 2–12 h, and without pre-infarction angina, bypass coronary intervention, and previous treatment with non-volatile anesthetic drugs.

Further, we are strongly confident that the correct timing of pharmacological and mechanical intervention application according to their specific ability to interfere with survival pathways is needed to significantly affect post-AMI LVR onset. In a population with the abovementioned clinical characteristics, we suggest the administration of RIPer-C, liraglutide (in the absence of hypoglycemia or diabetic ketoacidosis), and PCSK9 inhibitors (single “attack” dose of evolocumab 420 mg or alirocumab 450 mg) to the standard optimal therapy in the ‘pre-pPCI’ phase in order to initiate protection.

In the ‘during-pPCI’ phase, the finding of TIMI flow 0–1 at coronary angiography will be required to confirm patient enrollment, whereas STEMI patients with TIMI flow ≥2 will be excluded. Direct stenting will be the preferred technique in the case of partial coronary flow restoration after the wire crossing of the culprit lesion, otherwise a gentle pre-dilatation with a PCI-balloon will be needed. This fact will not determine patient withdrawal from the study, because this is a very common condition during pPCI. Post-dilatation with non-compliant PCI balloon will be allowed if the stent deployment is not satisfactory. A single protocol of RIPost-C will be used in an attempt to further extend the protective window provided by RIPer-C against MIRI. If a large thrombus burden is present, especially in hyperglycemic patients, TA will be performed; in the case of NR, Ade will be used. Both interventions will be left to the operator’s discretion.

In the ‘post-pPCI’ phase, the early administration of ARNI (immediately if naïve or after 36 h wash-out from RAAS modulators) and glifozins (except patients with hypoglycemia) will be used in order to prolong the window for cardiac protection and improve LVEF recovery.

The primary clinical endpoints will be the incidence of LVR and hospitalizations for new congestive HF at three months, six months, and one year-follow-up in a group of STEMI patients treated with the abovedescribed multitarget therapeutic strategy compared to a control group who will undergo classical optimal therapy according to international guidelines (pPCI + optimal pharmacological therapy). The secondary endpoint will be a composite of cardiac death, re-infarction, and new congestive HF at long-term follow-up (three years).

In the case of a demonstrated significant reduction in post-AMI LVR and HF incidence, the cost–benefit ratio of this strategy will also be evaluated on the basis of reduced mortality and re-hospitalizations for HF after long-term follow-up (three years).

## 5. Conclusions

The significant reduction in ‘ischemic time’ through capillary diffusion of pPCI rendered MIRI prevention a major issue in order to reduce the incidence of post-AMI LVR and improve the prognosis of STEMI patients. Single pharmacological and mechanical interventions have shown some benefits, but have not satisfactorily reduced mortality. Thus, a multitarget strategy is needed, but no univocal results have come from clinical studies performed so far. In this review, after a description of the pathogenic mechanisms that are responsible for the development of post-AMI LVR, we discussed the conventional and emerging pharmacological treatments, as well as the mechanical interventions, that have been shown to enhance cardioprotection. Finally, we tried to design a randomized clinical trial aimed at evaluating the effects of a reasoned multitarget therapeutic strategy on the prevention of post-AMI LVR and HF.

## Figures and Tables

**Table 1:** Principal miRs involved in post-AMI LVR phenomenon. For each miR, the actions on selective molecular targets, the determined physiological effects, and experimental models (organ/cell lines) that were used are indicated. Furthermore, drugs or mechanical interventions selectively modulating the expression of each miR are reported. miRs: micro-RNAs; AMI: acute myocardial infarction; LVR: left ventricular remodeling; Cx43: connexin43; TIMPs: tissue inhibitor of metalloproteinases; TGF-β: tumor growth factor-β; VCAM-1: vascular cell adhesion molecule-1; eNOS: endothelial nitric oxide synthase; MMPs: metalloproteinases; IS: infarct size; LV: left ventricle; ECM: extra-cellular matrix; EPCs: endothelial progenitor cells; RIPer-C: remote ischemic perconditioning; SAC/VAL: sacubitril/valsartan. ↑ indicates an increase; ↓ indicates a decrease.

miRs	Target	Physiological Effects	Model (Organ/Cell Line)	Drug and Mechanical Intervention	Drug Effects on miR Expression
miR-1	↓ Cx43 expression	↑ Arrhythmias	Rat (heart/cardiomyocytes, fibroblasts)	Metoprolol, RIPer-C	↓
miR-17	↓ TIMPs	↑ MMPs activity	Mouse (heart)	Not known	Not known
miR-21	↓ Apoptosis	↓ IS extension	Rat (heart/cardiomyocytes)	Not known	Not known
miR-24	↓ TGF-β, fibronectin, collagen type 1	↓ Fibrosis in area at risk	Mouse (heart/cardiomyocytes)	RIPer-C	↑
miR-126	↓ VCAM-1	↓ Plaque formation	Mouse (carotid)	Atorvastatin	↑
miR-181a	↓ Expression of TGF-β receptor III	↑ Fibrosis, hypertrophy	Rodent (Pluripotent stem cells, cardiomyocytes)	SAC/VAL	↓
miR-208	↑ Endoglin	↑ Cardiac fibrosis	Rat (heart)	Valsartan, Atorvastatin	↓
miR-210	↑ Micro-vessel density	↑ LV contractility	Rat (heart/cardiomyocytes)	Not known	Not known
miR-221/222	↓ eNOS activity	↓ Angiogenesis	Human (EPCs)	Atorvastatin	↓
miR-375	↓ PDK1, PI3K and Akt activity	↓ Cell survival	Mouse (heart/pancreatic β-cells)	Liraglutide	↓
miR-532	↓ prss activity	↓ Synthesis of ECM proteins	Mouse (heart)	Not known	Not known
Let-7c	↓ T cell proliferation	↓ Plaque rupture	Human (dendrocytes)	Atorvastatin	↑
Let-7i	↓ Toll-like receptor	↓ Activation of atherosclerotic plaque	Human (monocyte)	Atorvastatin	↑

## Data Availability

Not applicable.
